# Characterisation of body size phenotypes in a middle-aged Maltese population

**DOI:** 10.1017/jns.2021.74

**Published:** 2021-09-24

**Authors:** Rachel Agius, Nikolai Paul Pace, Stephen Fava

**Affiliations:** 1 Faculty of Medicine and Surgery, University of Malta, Tal-Qroqq, Msida, Malta; 2 Mater Dei Hospital, Triq Dun Karm, Msida, MSD 2090, Malta

**Keywords:** Body size phenotypes, Metabolic health, Obesity, Phenotype characterisation, Prevalence

## Abstract

Obesity is increasingly recognised as being a heterogeneous disease. Some obese individuals may present a metabolically healthy profile (metabolically healthy obese (MHO)), while some normal weight individuals exhibit an adverse cardiometabolic phenotype (metabolically unhealthy normal weight individuals (MUHNW)). The objectives of the present study were to examine the prevalence and associated characteristics of the different body composition phenotypes within a Maltese cohort. This was a cross-sectional analysis involving 521 individuals aged 41 ± 5 years. The metabolically unhealthy state was defined as the presence of ≥2 metabolic syndrome components (NCEP-ATPIII parameters), while individuals with ≤1 cardiometabolic abnormalities were classified as metabolically healthy. Overall, 70 % of the studied population was overweight or obese and 30⋅7 % had ≥2 cardiometabolic abnormalities. The prevalence of MHO and MUHNW was 10⋅7 and 2⋅1 %, respectively. Individuals with the healthy phenotype were more likely to consume alcohol, participate in regular physical activity and less likely to be smokers. While the MHO phenotype had similar values for waist, hip and neck circumferences, waist–hip ratio and insulin resistance when compared with MUHNW individuals, there was a lower proportion of MHO subjects having a high fasting plasma glucose, hypertriglyceridaemia or low HDL-C when compared with the unhealthy lean individuals. A high prevalence of the metabolically unhealthy phenotype was observed in this relatively young population which may result in significant future cardiovascular disease burden if timely assessment and management of modifiable risk factors are not implemented. Furthermore, the present study suggests that the MHO phenotype is not totally benign as previously thought.

## Introduction

Obesity is a well-established cardiovascular risk factor^(1)^. However, it is now also recognised that an individuals’ risk of cardiovascular disease does not solely depend on body size but also on their metabolic profile. Individuals with a similar body mass index (BMI) may exhibit different cardiometabolic risk parameters leading to a difference in the risk of cardiovascular disease. In order to better capture this variation, different body composition phenotypes have now been described taking into consideration both the body size (as expressed by the BMI) and the presence or absence of certain metabolic parameters.

At one end is the metabolically healthy normal weight individual (MHNW). This subset of individuals is characterised by a normal BMI (18⋅5 to <25 kg/m^2^) and the absence of any cardiometabolic risk profiles (including hypertension, dyslipidaemia and dysglycaemia). A second body size phenotype is the individual with normal weight but who harbours cardiometabolic abnormalities. This phenotype is termed as the metabolically unhealthy normal weight individual (MUHNW). Similarly, there are subjects who are overweight (BMI ≥ 25 to <30 kg/m^2^) or obese (BMI ≥30 kg/m^2^) and who may or may not harbour these adiposity-associated cardiometabolic abnormalities and thus lead to the occurrence of another four different body composition phenotypes: metabolically healthy overweight (MHOW), metabolically unhealthy overweight (MUHOW), metabolically healthy obese (MHO) and metabolically unhealthy obese phenotypes (MUHO)^(2)^.

Despite the importance of these phenotypes in predicting future cardiovascular risk, there is still incomplete data on their prevalence in various populations. The purpose of the present study was to determine the prevalence of the six different body size phenotypes in a Mediterranean population. The study also aimed to better characterise these body size phenotypes in terms of both cardiometabolic profile and lifestyle habits.

We conducted the present study in a cohort of middle-aged subjects so that the studied population would have lived long enough for phenotypic expression while reducing the risk of survival bias. Furthermore, sarcopenia is uncommon in this age group. Loss of muscle bulk results in a lower BMI for the degree of adiposity and this may contribute to the observation that the prevalence of MHO decreases with increasing age^(3)^. Sarcopenia may also result in insulin resistance (IR) since muscle is a major insulin-sensitive organ. The prevalence of the various body size phenotypes and the relationship between BMI and cardiometabolic health are therefore likely to be different in an elderly population, which should be studied separately.

### Research design and methods

This was an observational cross-sectional study carried out between January 2018 and June 2019, involving the recruitment of a nationally representative sample of a Maltese Caucasian non-institutionalized population aged 41 ± 5 years. We used a convenience type of sampling similar to that used in the ABCD study^(4)^. Exclusion criteria were subjects with a history of type 1 diabetes, individuals with known underlying genetic or endocrine causes of overweight or underweight (apart from controlled thyroid disorders), individuals with a terminal illness or active malignancy, individuals who could not give their own voluntary informed consent and pregnant females.

Participants were invited to attend for a one time visit, whereby demographic, health behaviour and lifestyle factors were captured via the use of a structured questionnaire especially designed for the survey. Level of education was stratified as primary, secondary or tertiary education according to the highest completed level. Occupation was coded into nine major categories as per the 1994 Spanish National classification of Occupations and thereafter re-classified into either white collar (non-manual) workers or blue collar (manual). Workers in the first four categories were deemed white collar workers, while those falling in the last five categories were grouped as blue collar workers, as previously described in the study by Sanchez-Chaparro *et al.*^(5)^. Smoking status, alcohol consumption and physical activity were classified as the National Health and Nutritional Examination Survey^(2)^.

### Anthropometric measurements

Anthropometric measurements were recorded with the participants dressed in light clothing and without shoes, using validated measurement equipment which was calibrated in accordance with WHO regulations. Body weight was measured in kilograms to the nearest 0⋅1 kg and height was measured in centimetres to 1 decimal place using a calibrated stadiometer with a vertical backboard and a movable headboard. BMI was calculated as a ratio of the weight (in kg) divided by the square of the height (in m). Participants were defined as having normal weight if the BMI value was <25 kg/m^2^; overweight if the BMI fell between the values 25⋅0–29⋅9 kg/m^2^ and obese if the BMI was ≥30 kg/m^2^. Waist and hip circumferences (WC and HC, respectively) were measured to the nearest 0⋅1 cm with a non-stretchable measuring tape over the abdomen halfway between the bottom of the rib cage and superior iliac crest for WC and over the widest diameter around the buttocks for the HC with the participants standing with their feet together such that weight was evenly distributed over both feet and after full expiration. Waist Index (WI) was calculated as WC (cm)/94 for men and WC (cm)/80 for females^(6)^. Other anthropometric parameters measured were neck circumference (NC), mid-upper arm circumference (MUAC) and thigh circumference (ThC). NC was measured at mid-neck height (to the nearest 1 mm) located by placing the measuring tape between the mid-cervical spine to mid-anterior neck^(7)^. The MUAC was identified by asking the participant to bend the elbow at a 90-degree angle, with the arm held parallel to the side of the body. Thereafter the midpoint of the distance between the acromion and olecranon process was identified and marked and the measuring tape was placed around this identified point for both arms. The upper ThC was measured by placing the tape over the largest portion of the thigh (at the level of the gluteal fold) with the thigh muscles fully relaxed and placed either directly over the skin or over very light clothing. Mean values for MUAC and ThC readings taken from the right and left sides are presented. All circumferences were taken with the subjects standing upright, with shoulders and thighs relaxed, facing the investigator^(8)^. Waist-to-height ratio (WHtR), waist-to-hip ratio (WHR), waist-to-thigh ratio (WThR) and arm-to-height ratio were calculated as WC (cm)/height (cm), WC (cm)/HC (cm), WC (cm)/ThC (cm) and MUAC/height, respectively. The conicity index (CI) was defined as waist/(0⋅109 × √weight (kg)/height (m))^(9)^ and the body adiposity index (BAI) as (HC/Height^2/3^) − 18, as previously described^(10)^. The atherogenic index of plasma (AIP) was calculated as (log(TG/HDL-C))^(11)^, while the lipid accumulation product (LAP) was calculated by the formulae (WC − 65) × (TG) in males and (WC − 58) × (TG) in females (WC in cm and TG in mmol/l)^(12)^. Body Roundness Index (BRI) was described by Thomas *et al.*^(13)^.

Blood pressure was measured according to the European Society of Hypertension Guidelines using a clinically validated digital sphygmomanometer with an appropriately sized cuff for each participant after a 5 min rest in the seated position; the average of the second and third readings was used for analyses^(14)^.

### Biochemical parameters

Fasting serum insulin was measured using a solid-phase sandwich enzyme linked immunosorbent assay (ELISA) (kit: Diagnostic Automation, USA). The homeostasis model assessment (HOMA) was used to evaluate IR using the formula: fasting serum insulin (microunits per millilitre) × fasting plasma glucose (millimoles per litre)/22⋅5^(15)^.

### Body size phenotype definitions

Body size phenotypes were generated based on the combined consideration of each participants’ BMI category as defined earlier and metabolic health. The metabolic syndrome components based on the National Cholesterol Education Program (NCEP) Adult Treatment Panel III (ATPIII) criteria were used to classify metabolic health as in previous studies^(16)^. This consisted of the following cardiometabolic (CM) parameters: waist circumference (WC) >102 cm in men and >88 cm in women; systolic/diastolic blood pressure (SBP/DBP) ≥130/85 mmHg or on antihypertensive medication; serum triglyceride level ≥1⋅69 mmol/l or on lipid-lowering medication; HDL-C <1⋅03 mmol/l in men and <1⋅29 mmol/l in women or on treatment aimed to increase HDL-C; fasting plasma glucose ≥5⋅6 mmol/l or on antihyperglycemic agents. Individuals were classified as being metabolically healthy if they exhibited 1 or less CM abnormalities from the above parameters in the first instance.

IR as measured by HOMA-IR was another criterion used to assess the prevalence of metabolic health in this population. A cut-off value of <2⋅5 was used to identify the metabolically healthy phenotype. This cut-off value has been chosen as it has already been validated in other studies^(17,18)^.

In the initial analyses, overweight and obese subjects were analysed together as one entity thus generating four body composition phenotypes: metabolically healthy normal weight (MHNW), metabolically unhealthy normal weight (MUHNW), metabolically healthy overweight or obese (MHOW/O) and metabolically unhealthy overweight or obese (MUHOW/O).

In secondary analyses, the overweight and obese subjects were analysed as separate entities thus generating two other body composition phenotypes in addition to the previous ones: MHOW (metabolically healthy overweight) and MUHOW (metabolically unhealthy overweight).

### Statistical analysis

Data are presented as medians and interquartile range for continuous variables and as percentages for categorical variables. Normality was assessed using the Shapiro–Wilk and Kolmogorov–Smirnov tests. All continuous variables showed a skewed non-normal distribution and non-parametric tests were used for comparisons. To evaluate differences in quantitative variables between groups, Kruskal–Wallis ANOVA was used for comparison between three or more categories, followed by Dunn's *post hoc* test was for pairwise comparison between subgroups. The independent samples Mann–Whitney *U* test was used for comparison between two categories. Bonferroni adjustment of *P*-values for multiple comparisons was applied. The *χ*^2^ test was used to compare categorical variables. All analysis was performed using IBM SPSS version 22. A *P-*value of <0⋅05 was considered significant.

### Ethical approval

This study was conducted in accordance with the 1964 Helsinki declaration and its later amendments and comparable ethical standards. All participants gave their written informed consent stating willingness to participate in this study as well as to undergo physical examination and biochemical testing. Ethical and data protection approvals were granted from the University of Malta Research Ethics Committee (UREC) of the Faculty of Medicine and Surgery and the Information and Data Protection Commissioner, respectively.

## Results

### Characteristics of the study population

A total of 521 individuals of Maltese ethnicity were assessed and provided the data for all the parameters required to define metabolic health status in the present study; 330 participants (63⋅3 %) were female. The median age was 41 years (range 30–51 years). The prevalence of the different BMI categories in the studied population was as follows: normal weight – 29⋅9 %, overweight – 36⋅7 % and obese – 33⋅3 %. Overall, 70 % of the study participants were either overweight or obese. The median weight was 78 kg (IQR 26), the median BMI was 27⋅5 kg/m^2^ (IQR 7⋅8) and the median WC was 89 cm (IQR 20). With respect to lifestyle characteristics, 22⋅5 % were active smokers (median 10 cigarettes per day) and 47⋅8 % regularly consumed alcohol (median 2 units per day). Just under a half of the participants (42⋅8 %) were physically active and 50 % achieved a tertiary level of education. Upon recruitment, 22 % (*n* 115) had a known medical comorbidity. These included type 2 diabetes (4⋅78 %), hypertension (7⋅84 %), hypothyroidism (4⋅2 %) and dyslipidaemia (6⋅11 %).

In this cohort of middle-aged subjects, the prevalence of unhealthy phenotype was 32⋅8 % (*n* 171), being made up of 30⋅7 % MUHOW/O and 2⋅1 % MUHNW. Healthy overweight/obesity (MHOW/O) was 39⋅3 % (*n* 205) (Table 1). A total of 56⋅1 % of overweight/obese individuals carried the MHO phenotype. Tables 1–4 show the demographic, biochemical and anthropometric characteristics of the study population according to the four different body composition phenotypes: MHNW, MUHNW, MHOW/O and MUHOW/O (incorporating the presence of 0–1 cm abnormalities of the NCEP-ATPIII criteria to define the metabolically healthy phenotype). Although a significant difference in age was not found between the healthy and unhealthy normal weight cohorts, a difference in age between the healthy and unhealthy overweight/obese subjects was noted with the MHO phenotype being slightly younger.

**Table 1.** Demographic characteristics of the study population according to BMI and metabolic status

	Metabolically healthy overweight/obese (MHOW/O)* *n* 205 (39⋅3 %)		Metabolically unhealthy overweight/obese (MUHOW/O)^†^ *n* 160 (30⋅7 %)		Metabolically healthy normal weight (MHNW)* *n* 145 (27⋅8 %)		Metabolically unhealthy normal weight (MUHNW)^†^ *n* 11 (2⋅11 %)		*P*-value^a^	*P*-value^b^	*P*-value^c^	*P*-value^d^
Age (median + IQR)	40⋅0	5⋅00	42⋅0	7 00	41⋅00	6⋅0	42⋅00	9⋅00	0⋅02	0⋅453	0⋅74	0⋅50
% Alcohol drinkers	53⋅2		37⋅5		53⋅8		18⋅2		0⋅01	0⋅06	0⋅484	0⋅03
% Smokers	17⋅6		26⋅9		24⋅1		27⋅3		0⋅025	0⋅554	0⋅235	0⋅258
% Regular physical activity	45⋅4		33⋅1		50⋅3		36⋅4		0⋅012	2⋅92	0⋅209	0⋅758
% White collar occupation	67⋅3		60		71⋅7		54⋅5		0⋅286	0⋅003	0⋅144	0⋅212
% PMH	11⋅7		40		13⋅1		54⋅5		0⋅01	0⋅003	0⋅408	<0⋅01
% Tertiary education	53⋅7		42⋅5		57⋅2		36⋅4		0⋅037	0⋅152	0⋅290	0⋅356

Data are expressed as a percentage or as median + IQR.

Abbreviations: BMI, body mass index; PMH, past medical history.

* Metabolically healthy – individuals having ≤1 NCEP-ATPIII criteria (consisting of waist circumference >102 cm in men and >88 cm in women; systolic or diastolic blood pressure ≥130/85 mmHg or on antihypertensive medication; serum triglycerides ≥1⋅69 mmol/l or on lipid-lowering medication; HDL-C <1⋅03 mmol/l in men and <1⋅29 mmol/l in women or on treatment aimed to increase HDL-C; fasting glucose ≥5⋅6 mmol/l or on antihyperglycemic agents).

† Metabolically unhealthy – individuals having ≥2 metabolic abnormalities of the NCEP-ATPIII criteria.

^a^*P*-value: MHOW/O *v*. MUHOW/O; ^b^*P*-value: MHNW *v*. MUHNW ^c^*P*-value; MHNW *v*. MHOW/O; ^d^*P*-value: MUHNW *v*. MHOW/O.

**Table 2.** Percentage of the study subjects having one or more metabolic syndrome components according to BMI and metabolic status

Metabolic syndrome components	Metabolically healthy overweight/obese (MHOW/O)* *n* 205 (39⋅3 %)	Metabolically unhealthy overweight/obese (MUHOW/O)^†^ *n* 160 (30⋅7 %)	Metabolically healthy normal weight (MHNW)* *n* 145 (27⋅8 %)	Metabolically unhealthy normal weight (MUHNW)^†^ *n* 11 (2⋅11 %)	*P*-value^a^	*P*-value^b^	*P*-value^c^	*P*-value^d^
WC ≥ 102 cm (M) or ≥88 cm (F)	27⋅8	78⋅8	2⋅1	72⋅7	<0⋅01	<0⋅01	<0⋅01	0⋅543
% FPG ≥ 5⋅6 mmol/l	7⋅3	54⋅4	4⋅8	54⋅5	<0⋅01	<0⋅01	0⋅237	0⋅01
% SBP ≥ 130 mmHg or DBP ≥85 mmHg or on antihypertensive agents	36⋅1	57⋅5	30⋅3	54⋅5	<0⋅01	0⋅096	0⋅157	0⋅336
% TG ≥ 1⋅7 mmol/l or on statins	7⋅8	51⋅2	0⋅7	63⋅6	<0⋅01	<0⋅01	<0⋅01	<0⋅01
% HDL-C ≤1⋅29 mmol/l (F) or ≤1⋅02 mmol/l (M) or on statins	8⋅3	63⋅1	9⋅7	63⋅6	<0⋅01	<0⋅01	0⋅398	<0⋅01

Data are expressed as percentages.

Abbreviations: BMI, body mass index; DBP, diastolic blood pressure; F, female; FPG, fasting plasma glucose; HDL-C, high density lipoprotein cholesterol; M, male; SBP, systolic blood pressure; TG, triglycerides; WC, waist circumference.

* Metabolically healthy – individuals having ≤1 NCEP-ATPIII criteria (consisting of waist circumference >102 cm in men and >88 cm in women; systolic or diastolic blood pressure ≥130/85 mmHg or on antihypertensive medication; serum triglycerides ≥1⋅69 mmol/l or on lipid-lowering medication; HDL-C <1⋅03 mmol/l in men and < 1⋅29 mmol/l in women or on treatment aimed to increase HDL-C; fasting glucose ≥5⋅6 mmol/l or on antihyperglycemic agents.

† Metabolically unhealthy – individuals having ≥2 metabolic abnormalities of the NCEP-ATPIII criteria.

^a^*P*-value: MHOW/O *v*. MUHOW/O; ^b^*P*-value: MHNW *v*. MUHNW; ^c^*P*-value: MHNW *v*. MHO; ^d^*P*-value: MUHNW *v.* MHO.

**Table 3.** Anthropometric parameters and indices of obesity measurement of the study subjects according to BMI and metabolic status

	Metabolically healthy overweight/obese (MHOW/O)* *n* 205 (39⋅3 %)		Metabolically unhealthy overweight/obese (MUHOW/O)^†^ *n* 160 (30⋅7 %)		Metabolically healthy normal weight (MHNW)* *n* 145 (27⋅8 %)		Metabolically unhealthy normal weight (MUHNW)^†^ *n* 11 (2⋅11 %)		*P-*value^a^	*P*-value^b^	*P*-value^c^	*P*-value^d^
	Median	IQR	Median	IQR	Median	IQR	Median	IQR				
Anthropometric parameters												
BMI (kg/m^2^)	27⋅8	4⋅2	32⋅9	7⋅5	22⋅4	2⋅6	24⋅0	1⋅6	<0⋅01	0⋅016	<0⋅01	<0⋅01
Waist circumference (cm)	89⋅0	13⋅0	103⋅0	13⋅2	74⋅0	11⋅0	82⋅0	15⋅0	<0⋅01	0⋅002	<0⋅01	0⋅19
Hip circumference (cm)	101⋅0	10⋅5	109⋅0	17⋅0	91⋅0	8⋅0	96⋅0	10⋅0	<0⋅01	0⋅021	<0⋅01	0⋅06
Neck circumference (cm)	36⋅0	5⋅0	38⋅3	6⋅5	31⋅0	2⋅5	33⋅0	8⋅0	<0⋅01	0⋅158	<0⋅01	0⋅08
Mean arm circumference (cm)	30⋅0	3⋅5	33⋅0	4⋅2	26⋅0	3⋅5	28⋅0	5⋅0	<0⋅01	0⋅031	<0⋅01	0⋅08
Mean thigh circumference (cm)	53⋅0	5⋅2	56⋅0	9⋅0	49⋅0	4⋅1	48⋅5	9⋅5	<0⋅01	0⋅479	<0⋅01	0⋅03
SBP (mmHg)	120⋅0	10⋅0	120⋅0	15⋅0	120⋅0	15⋅0	125⋅0	20⋅0	0⋅01	0⋅098	0⋅01	0⋅29
DBP (mmHg)	80⋅0	5⋅0	80⋅0	5⋅0	80⋅0	10⋅0	80⋅0	10⋅0	0⋅00	0⋅611	0⋅23	0⋅31
Indices of obesity measurement												
Visceral Adiposity Index	0⋅9	0⋅6	2⋅2	1⋅5	0⋅7	0⋅4	2⋅3	1⋅0	<0⋅01	<0⋅01	<0⋅01	<0⋅01
Waist Hip Ratio	0⋅9	0⋅1	0⋅9	0⋅1	0⋅8	0⋅1	0⋅9	0⋅1	<0⋅01	0⋅019	<0⋅01	0⋅76
Waist Height Ratio	0⋅5	0⋅1	0⋅6	0⋅1	0⋅5	0⋅1	0⋅5	0⋅0	<0⋅01	<0⋅01	<0⋅01	0⋅28
Waist Thigh Ratio	1⋅7	0⋅3	1⋅8	0⋅3	1⋅5	0⋅2	1⋅6	0⋅3	<0⋅01	<0⋅01	<0⋅01	0⋅86
Mean Arm Height Ratio	0⋅2	0⋅0	0⋅2	0⋅0	0⋅2	0⋅0	0⋅2	0⋅0	<0⋅01	0⋅073	<0⋅01	0⋅22
Body Adiposity Index	28⋅9	7⋅4	32⋅5	9⋅8	25⋅3	4⋅3	29⋅1	8⋅7	<0⋅01	0⋅112	<0⋅01	0⋅32
Conicity Index	1⋅2	0⋅1	1⋅3	0⋅1	1⋅1	0⋅1	1⋅2	0⋅1	<0⋅01	0⋅01	<0⋅01	0⋅03
Abdominal Volume Index	16⋅0	4⋅4	21⋅5	5⋅8	11⋅1	2⋅8	13⋅7	5⋅0	<0⋅01	0⋅02	<0⋅01	0⋅17
Body Roundness Index	3⋅8	1⋅4	5⋅8	1⋅9	2⋅5	0⋅9	3⋅7	0⋅9	<0⋅01	<0⋅01	<0⋅01	0⋅28
A Body Shape Index	0⋅1	0⋅0	0⋅1	0⋅0	0⋅1	0⋅0	0⋅1	0⋅0	<0⋅01	0⋅013	0⋅18	0⋅05
Waist Index	1⋅0	0⋅2	1⋅2	0⋅2	0⋅9	0⋅1	1⋅0	0⋅1	<0⋅01	0⋅01	<0⋅01	0⋅5

Data are expressed as median + IQR.

Abbreviations: BMI, body mass index; DBP, diastolic blood pressure; SBP, systolic blood pressure.

* Metabolically healthy – individuals having ≤1 NCEP-ATPIII criteria (consisting of waist circumference >102 cm in men and >88 cm in women; systolic or diastolic blood pressure ≥130/85 mmHg or on antihypertensive medication; serum triglycerides ≥1⋅69 mmol/l or on lipid-lowering medication; HDL-C <1⋅03 mmol/l in men and <1⋅29 mmol/l in women or on treatment aimed to increase HDL-C; fasting glucose ≥5⋅6 mmol/l or on antihyperglycemic agents.

† Metabolically unhealthy – individuals having ≥2 metabolic abnormalities of the NCEP-ATPIII criteria.

^a^*P-*value: MHOW/O *v*. MUHOW/O; ^b^*P*-value: MHNW *v*. MUHNW; ^c^*P*-value: MHNW *v*. MHO; ^d^*P*-value: MUHNW *v*. MHO.

**Table 4.** Biochemical parameters of the study subjects according to BMI and metabolic status

	Metabolically healthy overweight/obese (MHOW/O)* *n* 205 (39⋅3 %)		Metabolically unhealthy overweight/obese (MUHOW/O)^†^ *n* 160 (30⋅7 %)		Metabolically healthy normal weight (MHNW)* *n* 145 (27⋅8 %)		Metabolically unhealthy normal weight (MUHNW)^†^ *n* 11 (2⋅11 %)		*P*-value^a^	*P*-value^b^	*P*-value^c^	*P*-value^d^
	Median	IQR	Median	IQR	Median	IQR	Median	IQR				
Biochemical parameters												
Total cholesterol (mmol/l)	4⋅80	1⋅0	5⋅0	1⋅2	4⋅6	1⋅1	5⋅6	1⋅0	0⋅09	0⋅004	0⋅11	0⋅01
LDL-C (mmol/l)	2⋅8	1⋅0	3⋅1	1⋅2	2⋅6	1⋅0	3⋅7	0⋅7	0⋅01	<0⋅01	0⋅01	0⋅00
HDL-C (mmol/l)	1⋅5	0⋅4	1⋅2	0⋅3	1⋅7	0⋅5	1⋅2	0⋅1	<0⋅01	<0⋅01	<0⋅01	0⋅00
Triglycerides (mmol/l)	0⋅9	0⋅5	1⋅5	0⋅9	0⋅8	0⋅3	1⋅6	0⋅9	<0⋅01	<0⋅01	<0⋅01	<0⋅01
Uric acid	282⋅0	95⋅0	311⋅0	112⋅5	241⋅0	85⋅0	273⋅0	138⋅0	<0⋅01	0⋅222	<0⋅01	0⋅71
Fasting plasma glucose (mmol/l)	5⋅1	0⋅5	5⋅6	0⋅9	4⋅9	0⋅5	5⋅6	1⋅4	<0⋅01	0⋅234	0⋅00	0⋅50
HBA_1c_ (%)	5⋅2	0⋅4	5⋅5	0⋅7	5⋅2	0⋅3	5⋅3	0⋅2	<0⋅01	0⋅054	0⋅01	0⋅47
HOMA-IR	1⋅6	0⋅8	2⋅3	1⋅1	1⋅1	0⋅9	1⋅1	0⋅9	<0⋅01	0⋅563	<0⋅01	0⋅26
% HOMA-IR ≥2⋅5	6⋅6		45⋅6		3⋅4		22⋅2		<0⋅01	0⋅05	0⋅14	0⋅132
Vitamin D (ng/l)	18⋅0	9⋅0	17⋅0	6⋅5	19⋅0	10⋅0	15⋅0	4⋅0	0⋅03	0⋅032	0⋅26	0⋅05
ALP (U/l)	63⋅0	20⋅0	69⋅0	19⋅0	55⋅0	20⋅0	63⋅0	13⋅0	0⋅00	0⋅097	<0⋅01	0⋅83
GGT (U/l)	18⋅0	16⋅0	27⋅0	21⋅0	14⋅0	9⋅0	17⋅0	46⋅0	<0⋅01	0⋅037	<0⋅01	0⋅63
ALT (U/l)	16⋅0	13⋅0	22⋅0	16⋅0	14⋅0	9⋅0	18⋅0	30⋅0	<0⋅01	0⋅355	<0⋅01	0⋅92
Ferritin (ng/ml)	52⋅0	107⋅0	83⋅5	149⋅5	28⋅0	55⋅0	85⋅0	120⋅0	0⋅02	0⋅012	<0⋅01	0⋅47
TSH (micIU/ml)	1⋅5	0⋅9	1⋅5	1⋅1	1⋅5	1⋅2	1⋅3	1⋅3	0⋅15	1⋅000	0⋅50	0⋅86
FT4 (pmol/l)	14⋅7	2⋅5	14⋅5	2⋅7	14⋅7	2⋅3	14⋅8	2⋅9	0⋅18	0⋅563	0⋅37	0⋅33
LAP	26⋅2	19⋅5	63⋅4	44⋅7	11⋅6	9⋅0	34⋅4	30⋅7	<0⋅01	<0⋅01	<0⋅01	0⋅06
log(TG/HDL-C)	-0⋅2	0⋅3	0⋅1	0⋅4	−0⋅4	0⋅2	0⋅1	0⋅3	<0⋅01	<0⋅01	<0⋅01	<0⋅01
PLR	134⋅5	54⋅4	122⋅9	63⋅8	142⋅9	54⋅8	121⋅7	78⋅7	0⋅03	0⋅085	0⋅10	0⋅22
NLR	2⋅0	0⋅9	2⋅0	0⋅9	2⋅1	1⋅0	1⋅9	1⋅0	0⋅36	0⋅063	0⋅30	0⋅92

Data are expressed as median + IQR or percentages.

Abbreviations: ALP, alkaline phosphatase; ALT, alanine transaminase; BMI, body mass index; DBP, diastolic blood pressure; F, female; FPG, fasting plasma glucose; FT4, free thyroxine; GGT, Gamma glutamyl transferase; HBA_1c_, haemoglobin A1c; HDL-C, high density lipoprotein cholesterol; HOMA-IR, homeostatic model assessment of insulin resistance; LAP, lipid accumulation product; LDL-C, low density lipoprotein cholesterol; M, male; NLR, neutrophil lymphocyte ratio; PLR, platelet lymphocyte ratio; PMH, past medical history; SBP, systolic blood pressure; TG, triglycerides; TSH, thyroxine stimulating hormone; WC, waist circumference.

* Metabolically healthy – individuals having ≤1 NCEP-ATPIII criteria (consisting of waist circumference >102 cm in men and >88 cm in women; systolic or diastolic blood pressure ≥130/85 mmHg or on antihypertensive medication; serum triglycerides ≥1⋅69 mmol/l or on lipid-lowering medication; HDL-C <1⋅03 mmol/l in men and <1⋅29 mmol/l in women or on treatment aimed to increase HDL-C; fasting glucose ≥5⋅6 mmol/l or on antihyperglycemic agents.

† Metabolically unhealthy – individuals having ≥2 metabolic abnormalities of the NCEP-ATPIII criteria.

^a^*P*-value: MHOW/O *v*. MUHOW/O; ^b^*P*-value: MHNW *v*. MUHNW; ^c^*P*-value: MHNW *v*. MHO; ^d^*P*-value: MUHNW *v*. MHO.

Significant differences in baseline characteristics of overweight/obese subjects were found according to the presence or absence of metabolic syndrome. With respect to lifestyle factors, subjects with the MHOW/O phenotype were more likely to drink alcohol, engage in regular physical activity and have a higher level of education when compared with their metabolically unhealthy counterparts (MUHOW/O) (Table 1). On the other hand, the MHOW/O subjects had lower values for indices of obesity measurement compared with MUHOW/O including BMI (*P* < 0⋅01), WHR (*P* = 0⋅01), WI (*P* < 0⋅01), WHtR (*P* < 0⋅01), WThR (*P* < 0⋅01), VAI (*P* < 0⋅01), BAI (*P* < 0⋅01), CI (*P* < 0⋅01), AVI (*P* < 0⋅01), BRI (*P* < 0⋅001), ABSI (*P* < 0⋅01) and lower values for certain cardiometabolic risk factors (including FPG, LDL-C, TG, HBA_1c_ and HOMA-IR) but a higher HDL-C value as expected (Tables 3 and 4).

The prevalence of the metabolic unhealthy phenotype was 7 % among normal weight subjects (MUHNW). MUHNW subjects were more likely to have a non-manual (white collar) occupation and exhibit a current medical comorbidity. However, there were no other significant differences in terms of lifestyle characteristics when compared with their healthy counterparts. With respect to anthropometric parameters, the MUHNW phenotype had higher values for indices of central obesity measurement (including WI (*P* = 0⋅001), HC (*P* = 0⋅021), WHR (*P* = 0⋅019), WHtR (*P* = 0⋅001), WThR (*P* = 0⋅005), CI (*P* = 0⋅001), AVI (*P* = 0⋅002), BRI (*P* ≤ 0⋅001) and ABSI (*P* = 0⋅013)), but not for BAI (*P* = 0⋅112) (Table 3). Moreover, while the MUHNW subjects had significantly higher values for all lipid parameters, there were no differences between values for glycaemic parameters (FPG (*P* = 0⋅234), HBA_1c_ (*P* = 0⋅054) and HOMA-IR (*P* = 0⋅5)) when compared with the MHNW participants (Table 4).

The MHOW/O participants were of similar age to their healthy non-obese counterparts (MHNW) and were also comparable for several lifestyle variables including age, smoking and alcohol consumption, physical activity, presence of an underlying comorbidity as well as level of education and occupation (Table 1). Moreover, the proportions of individuals exhibiting one of the parameters of the metabolic syndrome except for triglycerides (*P* = 0⋅001) were similar to MHOW/O and MHNW (Table 2). However, subjects with the MHOW/O phenotype displayed higher values for all indices of obesity measurement, had higher lipid parameter values and were more insulin-resistant than the healthy non-obese individuals (MHNW; Tables 3 and 4). On the other hand, when the healthy overweight/obese phenotype was compared with the unhealthy normal weight subgroup, individuals within the MUHNW phenotype were less likely to drink alcohol but more likely to have a concomitant medical problem than the MHOW/O subjects. Such individuals had higher values for total cholesterol (TChol) and TG but similar values for IR as assessed by HOMA (Tables 1 and 4).

Tables 5–8 show the demographic, metabolic and anthropometric characteristics of the study population when stratified by the three different BMI categories (normal weight (BMI < 25 kg/m^2^), overweight (BMI 25–29⋅9 kg/m^2^) and obese (BMI > 30 kg/m^2^)) and metabolic health (adopting the presence of 0–1 NCEP-ATP III criteria to characterise the metabolically healthy phenotype) and BMI. In these tables, the obese cohort was subdivided into overweight (BMI 25–29⋅9 kg/m^2^) and obese (BMI > 30 kg/m^2^), thus generating the six different body composition phenotypes (MHNW, MHOW, MHO, MUHNW, MUHOW and MUHO). The population prevalence of each body composition phenotype was as follows: MHNW – 27⋅8 %, MHOW – 28⋅6 %, MHO – 10⋅7 %, MUHNW – 2⋅1 %, MUHOW – 8⋅1 % and MUHO – 22⋅6 %. 72⋅6 % (*n* 149) of the healthy overweight/obese cohort were characterised as MHOW, and 78 % of the total overweight cohort were metabolically healthy. On the other hand, within the MUHO cohort, 73 % (*n* 118) were metabolically unhealthy obese and only 26 % (*n* 42) were of the MUHOW phenotype (Table 5).

**Table 5.** Demographic characteristics of the study subjects by body size phenotype

			Metabolically healthy*						Metabolically Unhealthy^†^					
	Overall (*n* 521)		Normal weight *n* 145 (27⋅8 %)		Overweight *n* 149 (28⋅6 %)		Obese *n* 56 (10⋅7 %)		Normal weight *n* 11 (2⋅1 %)		Overweight *n* 42 (8⋅1 %)		Obese *n* 118 (22⋅6 %)	
Age	41⋅0	6⋅0	41⋅0	6⋅0	40⋅00	6⋅0	40⋅0	5⋅5	42⋅0	9⋅00	43⋅0	6⋅0	41⋅0	6⋅0
% Males	36⋅7		17⋅9 %		45⋅6 %		32⋅1 %		27⋅3 %		57⋅1 %		44⋅1 %	
% Alcohol drinkers	47⋅8		53⋅8 %		57⋅0 %		42⋅9 %		18⋅2 %		47⋅6 %		33⋅9 %	
% Smokers	22⋅6		24⋅1 %		15⋅4 %		23⋅2 %		27⋅3 %		28⋅6 %		26⋅3 %	
% Regular physical activity	42⋅8		50⋅3 %		49⋅0 %		35⋅7 %		36⋅4 %		45⋅2 %		28⋅8 %	
% White collar occupation	66⋅0		71⋅7 %		69⋅8 %		60⋅7 %		54⋅5 %		61⋅9 %		59⋅3 %	
% PMH	21⋅7		13⋅1 %		12⋅1 %		10⋅7 %		54⋅5 %		35⋅7 %		41⋅5 %	
% Tertiary education	50⋅9		57⋅2 %		53⋅0 %		55⋅4 %		36⋅4 %		45⋅2 %		41⋅5 %	

Data are expressed as a percentage or as median + IQR.

Abbreviations: BMI, body mass index; PMH, past medical history.

* Metabolically healthy – individuals having ≤1 NCEP-ATPIII criteria (consisting of waist circumference >102 cm in men and >88 cm in women; systolic or diastolic blood pressure ≥130/85 mmHg or on antihypertensive medication; serum triglycerides ≥1⋅69 mmol/l or on lipid-lowering medication; HDL-C <1⋅03 mmol/l in men and <1⋅29 mmol/l in women or on treatment aimed to increase HDL-C; fasting glucose ≥5⋅6 mmol/l or on antihyperglycemic agents).

† Metabolically unhealthy – individuals having ≥2 metabolic abnormalities of the NCEP-ATPIII criteria.

**Table 6.** Percentage of the study subjects having one or more metabolic syndrome components as stratified by body size phenotype

		Metabolically healthy*			Metabolically Unhealthy^†^		
	Overall (*n* 521)	Normal weight *n* 145 (27⋅8 %)	Overweight *n* 149 (28⋅6 %)	Obese *n* 56 (10⋅7 %)	Normal weight *n* 11 (2⋅1 %)	Overweight *n* 42 (8⋅1 %)	Obese *n* 118 (22⋅6 %)
Metabolic syndrome components (%)							
%WC ≥102 cm (M) or ≥88 cm (F)	36⋅3	2⋅1 %	14⋅8 %	62⋅5 %	27⋅3 %	45⋅2 %	90⋅7 %
% FBG ≥ 5⋅6 mmol/l	22⋅1	4⋅8 %	9⋅4 %	1⋅8 %	54⋅5 %	59⋅5 %	52⋅5 %
%HOMA-IR ≥ 2⋅5	18⋅1	3⋅4 %	7⋅7 %	3⋅7 %	22⋅2 %	40⋅5 %	47⋅4 %
% SBP ≥ 130 mmHg or DBP ≥85 mmHg or on antihypertensive agents	41⋅5	30⋅3 %	34⋅9 %	39⋅2 %	54⋅5 %	57⋅1 %	57⋅6 %
% TG ≥ 1⋅7 mmol/l or on statins	20⋅3	0⋅7 %	9⋅4 %	3⋅6 %	63⋅6 %	59⋅5 %	48⋅3 %
% HDL-C ≤1⋅29 mmol/l (F) or ≤1⋅02 mmol/l (M) or on statins	26⋅7	9⋅7 %	8⋅7 %	7⋅1 %	63⋅6 %	52⋅4 %	66⋅9 %

Data are expressed as percentages.

Abbreviations: DBP, diastolic blood pressure; F, female; FPG, fasting plasma glucose; HDL-C, high density lipoprotein cholesterol; M, male; SBP, systolic blood pressure; TG, triglycerides; WC, waist circumference.

* Metabolically healthy – individuals having ≤1 NCEP-ATPIII criteria (consisting of waist circumference >102 cm in men and >88 cm in women; systolic or diastolic blood pressure ≥130/85 mmHg or on antihypertensive medication; serum triglycerides ≥1⋅69 mmol/l or on lipid-lowering medication; HDL-C <1⋅03 mmol/l in men and <1⋅29 mmol/l in women or on treatment aimed to increase HDL-C; fasting glucose ≥5⋅6 mmol/l or on antihyperglycemic agents.

† Metabolically unhealthy – individuals having ≥2 metabolic abnormalities of the NCEP-ATPIII criteria.

**Table 7.** Anthropometric parameters and indices of obesity measurement of the study subjects by body size phenotype

			Metabolically healthy*						Metabolically unhealthy^†^					
	Overall (*n* 521)		Normal weight *n* 145 (27⋅8 %)		Overweight *n* 149 (28⋅6 %)		Obese *n* 56 (10⋅7 %)		Normal weight *n* 11 (2⋅1 %)		Overweight *n* 42 (8⋅1 %)		Obese *n* 118, (22⋅6 %)	
	Median	IQR	Median	IQR	Median	IQR	Median	IQR	Median	IQR	Median	IQR	Median	IQR
Anthropometric parameters														
BMI (kg/m^2^)	27⋅5	7⋅8	22⋅4	2⋅6	27⋅1	2⋅1	33⋅0	4⋅1	24⋅0	1⋅6	28⋅2	2⋅2	34⋅7	6⋅7
Waist circumference (cm)	89⋅0	20⋅0	74⋅0	11⋅0	86⋅5	11⋅0	96⋅0	12⋅0	82⋅0	15⋅0	92⋅0	10⋅0	106⋅0	12⋅5
Hip circumference (cm)	99⋅0	16⋅0	91⋅0	8⋅0	98⋅0	10⋅0	110⋅5	13⋅0	96⋅0	10⋅0	101⋅5	7⋅0	114⋅0	16⋅0
Neck circumference (cm)	35⋅0	6⋅0	31⋅0	2⋅5	35⋅6	5⋅0	36⋅0	5⋅5	33⋅0	8⋅0	38⋅0	8⋅0	40⋅0	6⋅0
Mean arm circumference (cm)	30⋅0	5⋅5	26⋅0	3⋅5	29⋅2	3⋅0	31⋅0	2⋅7	28⋅0	5⋅0	30⋅5	4⋅0	33⋅0	4⋅0
Mean thigh circumference (cm)	52⋅0	7⋅5	49⋅0	4⋅1	52⋅0	7⋅0	56⋅5	5⋅0	48⋅5	9⋅5	51⋅3	6⋅5	58⋅0	7⋅5
Average Arm to Height	0⋅2	0⋅0	0⋅2	0⋅0	0⋅2	0⋅1	0⋅2	0⋅0	0⋅2	0⋅0	0⋅2	0⋅0	0⋅2	0⋅0
SBP (mmHg)	120⋅0	10⋅0	120⋅0	15⋅0	120⋅0	10⋅0	122⋅5	8⋅5	125⋅0	20⋅0	125⋅0	10⋅0	120⋅0	15⋅0
DBP (mmHg)	80⋅0	10⋅0	80⋅0	10⋅0	80⋅0	10⋅0	80⋅0	5⋅0	80⋅0	10⋅0	80⋅0	5⋅0	80⋅0	5⋅0
Indices of central obesity measurement														
Visceral Adiposity Index	1⋅1	1⋅1	0⋅7	0⋅4	1⋅0	0⋅7	0⋅9	0⋅4	2⋅3	1⋅0	2⋅1	1⋅4	2⋅2	1⋅5
Waist Height Ratio	0⋅5	0⋅1	0⋅5	0⋅1	0⋅5	0⋅1	0⋅6	0⋅1	0⋅5	0⋅0	0⋅6	0⋅0	0⋅6	0⋅1
Waist Thigh Ratio	1⋅7	0⋅4	1⋅5	0⋅2	1⋅7	0⋅3	1⋅7	0⋅3	1⋅6	0⋅3	1⋅8	0⋅3	1⋅8	0⋅4
Body Adiposity Index	27⋅9	8⋅1	25⋅3	4⋅3	27⋅1	6⋅4	35⋅0	10⋅1	29⋅1	8⋅7	28⋅0	6⋅5	35⋅6	9⋅8
Conicity Index	1⋅2	0⋅1	1⋅1	0⋅1	1⋅2	0⋅1	1⋅2	0⋅1	1⋅2	0⋅1	1⋅3	0⋅1	1⋅3	0⋅1
Abdominal Volume Index	16⋅0	7⋅3	11⋅1	2⋅8	15⋅2	3⋅3	19⋅1	4⋅5	13⋅7	5⋅0	17⋅2	3⋅6	22⋅8	5⋅1
Body Roundness Index	3⋅9	2⋅2	2⋅5	0⋅9	3⋅7	0⋅9	5⋅1	1⋅5	3⋅7	0⋅9	4⋅5	0⋅9	6⋅2	1⋅9
A Body Shape Index	0⋅1	0⋅0	0⋅1	0⋅0	0⋅1	0⋅0	0⋅1	0⋅0	0⋅1	0⋅0	0⋅1	0⋅0	0⋅1	0⋅0
Waist Hip Ratio	0⋅9	0⋅1	0⋅8	0⋅1	0⋅9	0⋅1	0⋅9	0⋅1	0⋅9	0⋅1	0⋅9	0⋅1	0⋅9	0⋅2
Waist Index	1⋅0	0⋅2	0⋅9	0⋅1	1⋅0	0⋅1	1⋅2	0⋅2	1⋅0	0⋅1	1⋅0	0⋅2	1⋅2	0⋅2
Body Roundness Index	3⋅9	2⋅2	2⋅5	0⋅9	3⋅7	0⋅9	5⋅1	1⋅5	3⋅7	0⋅9	4⋅5	0⋅9	6⋅2	1⋅9
A Body Shape Index	0⋅1	0⋅0	0⋅1	0⋅0	0⋅1	0⋅0	0⋅1	0⋅0	0⋅1	0⋅0	0⋅1	0⋅0	0⋅1	0⋅0
Waist Hip Ratio	0⋅9	0⋅1	0⋅8	0⋅1	0⋅9	0⋅1	0⋅9	0⋅1	0⋅9	0⋅1	0⋅9	0⋅1	0⋅9	0⋅2
Waist Index	1⋅0	0⋅2	0⋅9	0⋅1	1⋅0	0⋅1	1⋅2	0⋅2	1⋅0	0⋅1	1⋅0	0⋅2	1⋅2	0⋅2

Data are expressed as median + IQR.

Abbreviations: DBP, diastolic blood pressure; SBP, systolic blood pressure.

* Metabolically healthy – individuals having ≤1 NCEP-ATPIII criteria (consisting of waist circumference >102 cm in men and >88 cm in women; systolic or diastolic blood pressure ≥130/85 mmHg or on antihypertensive medication; serum triglycerides ≥1⋅69 mmol/l or on lipid-lowering medication; HDL-C <1⋅03 mmol/l in men and <1⋅29 mmol/l in women or on treatment aimed to increase HDL-C; fasting glucose ≥5⋅6 mmol/l or on antihyperglycemic agents.

† Metabolically unhealthy – individuals having ≥2 metabolic abnormalities of the NCEP-ATPIII criteria.

**Table 8.** Biochemical parameters of the study subjects by body size phenotype

			Metabolically healthy*						Metabolically unhealthy^†^					
	Overall (*n* 521)		Normal weight *n* 145 (27⋅8 %)		Overweight *n* 149 (28⋅6 %)		Obese *n* 56 (10⋅7 %)		Normal weight *n* 11 (2⋅1 %)		Overweight *n* 42 (8⋅1 %)		Obese *n* 118 (22⋅6 %)	
	Median	IQR	Median	IQR	Median	IQR	Median	IQR	Median	IQR	Median	IQR	Median	IQR
Biochemical parameters														
TChol (mmol/l)	4⋅9	1⋅1	4⋅6	1⋅1	4⋅8	1⋅0	4⋅9	0⋅9	5⋅6	1⋅0	5⋅2	1⋅5	4⋅9	1⋅2
LDL-C (mmol/l)	2⋅9	1⋅1	2⋅6	1⋅0	2⋅8	0⋅9	2⋅9	0⋅9	3⋅7	0⋅7	3⋅1	1⋅2	3⋅1	1⋅2
HDL-C (mmol/l)	1⋅4	0⋅5	1⋅7	0⋅5	1⋅5	0⋅4	1⋅6	0⋅4	1⋅2	0⋅1	1⋅2	0⋅4	1⋅2	0⋅3
Triglycerides (mmol/l)	1⋅0	0⋅7	0⋅8	0⋅3	0⋅9	0⋅6	0⋅9	0⋅4	1⋅6	0⋅9	1⋅6	1⋅3	1⋅5	0⋅8
Uric Acid (umol/l)	280⋅0	95⋅0	241⋅0	85⋅0	280⋅0	90⋅0	286⋅0	94⋅0	273⋅0	138⋅0	308⋅5	120⋅0	311⋅5	113⋅0
FPG (mmol/l)	5⋅1	0⋅7	4⋅9	0⋅5	5⋅1	0⋅5	5⋅0	0⋅4	5⋅6	1⋅4	5⋅6	0⋅8	5⋅6	0⋅9
HBA_1c_ (%)	5⋅3	0⋅4	5⋅2	0⋅3	5⋅2	0⋅4	5⋅3	0⋅3	5⋅3	0⋅2	5⋅4	0⋅5	5⋅6	0⋅7
HOMA-IR	1⋅7	1⋅2	1⋅1	0⋅9	1⋅6	0⋅9	1⋅5	0⋅9	1⋅1	0⋅9	2⋅2	1⋅6	2⋅4	1⋅1
Vitamin D (ng/l)	18⋅0	8⋅0	19⋅0	10⋅0	18⋅0	10⋅0	18⋅0	7⋅5	15⋅0	4⋅0	17⋅0	6⋅0	18⋅0	7⋅0
ALP (U/l)	63⋅0	21⋅0	55⋅0	20⋅0	63⋅0	22⋅0	63⋅5	15⋅5	63⋅0	13⋅0	67⋅5	15⋅0	70⋅0	18⋅0
GGT (U/l)	19⋅0	16⋅0	14⋅0	9⋅0	18⋅0	16⋅0	18⋅0	14⋅5	17⋅0	46⋅0	29⋅0	26⋅0	25⋅0	18⋅0
ALT (U/l)	17⋅0	13⋅0	14⋅0	9⋅0	17⋅0	13⋅0	16⋅0	14⋅5	18⋅0	30⋅0	23⋅0	23⋅0	22⋅0	16⋅0
Ferritin (ng/ml)	53⋅0	105⋅0	28⋅0	55⋅0	52⋅0	112⋅0	52⋅0	84⋅5	85⋅0	120⋅0	77⋅5	175⋅0	85⋅0	147⋅0
TSH (micIU/ml)	1⋅5	1⋅0	1⋅5	1⋅2	1⋅4	0⋅8	1⋅7	1⋅2	1⋅3	1⋅3	1⋅5	0⋅9	1⋅5	1⋅1
FT4 (pmol/l)	14⋅6	2⋅5	14⋅7	2⋅3	15⋅1	2⋅6	14⋅4	2⋅1	14⋅8	2⋅9	14⋅7	2⋅2	14⋅4	2⋅8
LAP	27⋅5	36⋅5	11⋅6	9⋅0	23⋅7	18⋅4	33⋅4	22⋅1	34⋅4	30⋅7	52⋅0	35⋅6	65⋅9	45⋅9
log(TG/HDL-C)	−0⋅2	0⋅4	−0⋅4	0⋅2	−0⋅2	0⋅3	−0⋅3	0⋅3	0⋅1	0⋅3	0⋅1	0⋅4	0⋅1	0⋅3
PLR	134⋅4	62⋅3	142⋅9	54⋅8	135⋅2	56⋅7	127⋅6	54⋅1	121⋅7	78⋅7	127⋅8	70⋅5	119⋅8	65⋅3
NLR	2⋅0	0⋅9	2⋅1	1⋅0	1⋅9	0⋅8	2⋅0	1⋅0	1⋅9	1⋅0	1⋅9	0⋅6	2⋅1	1⋅0

Data are expressed as median + IQR.

Abbreviations: ALP, alkaline phosphatase; ALT, alanine transaminase; BMI, body mass index; DBP, diastolic blood pressure; F, female; FPG, fasting plasma glucose; FT4, free thyroxine; GGT, Gamma glutamyl transferase; HBA_1c_, haemoglobin A1c; HDL-C, high density lipoprotein cholesterol; HOMA-IR, homeostatic model assessment of insulin resistance; LAP, lipid accumulation product; LDL-C, low density lipoprotein cholesterol; M, male; NLR, neutrophil lymphocyte ratio; PLR, platelet lymphocyte ratio; PMH, past medical history; SBP, systolic blood pressure; TG, triglycerides; TSH, thyroxine stimulating hormone; WC, waist circumference.

* Metabolically healthy – individuals having ≤1 NCEP-ATPIII criteria (consisting of waist circumference >102 cm in men and >88 cm in women; systolic or diastolic blood pressure ≥130/85 mmHg or on antihypertensive medication; serum triglycerides ≥1⋅69 mmol/l or on lipid-lowering medication; HDL-C <1⋅03 mmol/l in men and <1⋅29 mmol/l in women or on treatment aimed to increase HDL-C; fasting glucose ≥5⋅6 mmol/l or on antihyperglycemic agents.

† Metabolically unhealthy – individuals having ≥2 metabolic abnormalities of the NCEP-ATPIII criteria.

Overall, the metabolically healthy phenotype was more prevalent in women, in those with a tertiary level of education and in those holding a white collar (non-manual) occupation. There was a lower proportion of individuals within the overweight and obese categories who engaged in some regular form of physical activity (Table 1). As expected, there was a trend towards increasing values in all indices of obesity measurement (WC, BMI, WI, WThR, WHtR, BAI, CI, AVI, BRI and ABSI) as well as certain biochemical parameters (TChol, LDL-C and HDL-C) from healthy normal weight to obesity state (Tables 7 and 8).

Within the unhealthy group, obesity was also associated with the female sex and with a lower likelihood of engaging in physical activity. Similarly, there was also a significant trend towards an increase in values of certain anthropometric parameters from normal weight to obese BMI categories (including WC, HC, NC, AC, ThC, WI, WHR, WHtR, AVI and BRI), and for most indices of obesity measurement, however this trend was not observed within certain biochemical parameters including lipid profile and fasting glucose levels (Tables 7 and 8).

Of note, 7⋅7 % of overweight subjects without Met-S were insulin-resistant but only 3⋅7 % of metabolically healthy obese individuals were insulin-resistant as evident by the proportion of subjects having a HOMA-IR value of ≥2⋅5. On the other hand, 22 % of normal weight subjects with Met-S were insulin-resistant and nearly half of the obese subjects with Met-S were insulin-resistant (Table 6).

## Discussion

In our population with a mean age of 40 years, almost one third (30⋅7 %) were metabolically unhealthy using the presence of at least 1 NCEP-ATP III criteria as the cut-off. Most of these were obese (22⋅6 %) or overweight (8⋅1 %), but a few (2⋅1 %) were of normal weight. Use of more stringent criteria would have resulted in an even larger prevalence of the metabolic unhealthy phenotype. This high prevalence in such a relatively young population would be expected to result in an increased future cardiovascular disease burden. The prevalence of obesity and of smoking in our sample is similar to that reported in the Maltese general population (33⋅3 *v*. 34⋅1 % and 22⋅5 *v*. 24⋅3 %, respectively)^(19,20)^. However, the prevalence of diabetes and hypertension was less, reflecting the age range of our cohort (4⋅2 *v.* 10⋅4 % and 7⋅8 *v*. 30⋅1 %, respectively)^(20,21)^.

There is a wide variation in the reported prevalence of MHO ranging between 6 and 75 %^(22)^. In our cohort of middle-aged subjects, the prevalence of healthy overweight/obesity was 39⋅3 %, which is more than double that the 15⋅1 % reported in Spain^(23)^, another Mediterranean country. This may be related to a more rapidly declining adherence to the Mediterranean diet in Spain compared with Malta^(24,25)^, differences in age distribution between the two cohorts or to possible differences in genetic ancestry between the two countries. Contemporary Maltese originated from settlers in Sicily and Southern Italy and the subsequent turbulent history characterised by multiple conquests, immigration and depopulation have affected the genetic legacy of the Maltese. Conversely, a larger degree of genetic admixture within Spanish populations can be explained by different historical trajectories^(26,27)^. In this context, regional differences in chronic disease prevalence can be partially attributed to variation in population genetics.

As expected, the metabolically healthy subjects had more favourable anthropometric and biochemical parameters than their metabolically unhealthy counterparts irrespective of whether they were of normal weight or overweight/obese. They had higher HDL and lower triglycerides and LAP. The latter is an index of central lipid accumulation and of visceral obesity and has been used to predict the risk of metabolic syndrome and subclinical atherosclerosis and cardiovascular risk^(12)^.

In spite of the fact that BMI was used to categorise individuals into being normal weight or overweight/obese, the healthy individuals still had a lower BMI within their respective categories. They also had a lower WHR, WHtR and WThR, which are all markers of visceral adiposity. The WHR has also been suggested to be a negative marker of reverse cholesterol transfer^(28)^.

Metabolically healthy individuals in both the normal weight and overweight/obese categories also had lower neck circumference, confirming the utility of this measure. Other authors have reported that neck circumference compares favourably with an abdominal circumference in predicting cardiometabolic abnormalities and is correlated with IR and the metabolic syndrome^(29,30)^.

BAI, visceral adiposity index (VAI), CI, abdominal volume index (AVI), BRI and ‘A’ body shape index (ABSI) were also lower in the metabolically healthy subjects irrespective of whether they were of normal weight or obese/overweight. The BAI is a marker of total body fat^(10)^, while the other indices are markers of visceral fat^(13,31,32)^. These anthropometric markers have been shown to predict cardiovascular disease^(33,34)^.

In the overweight/obese group, TChol was lower in the metabolically healthy, but the opposite was true in the normal weight group. This may be due to the fact that TChol reflects both subcutaneous fat and muscle mass^(34)^. In the overweight/obesity group, ThC may be a better indicator of generalised adiposity. It is noteworthy that many of the studies reporting low ThC to be associated with adverse cardiometabolic outcomes were done in relatively lean individuals^(34,35)^.

Arm circumference has been proposed as a positive marker of metabolic health^(36,37)^. We could not confirm this in our cohort since metabolically healthy individuals had smaller arm circumference to metabolically unhealthy ones. It is, therefore, likely that there may be population differences between the relationships of arm circumference and cardiometabolic health. This may be driven by population differences in the relative contribution of fat and muscle to this measure. Furthermore, the arm-to-height ratio did not perform any better than the arm circumference in predicting individual's cardiometabolic health.

We found that metabolically healthy individuals were more physically active than metabolically unhealthy individuals. This was true in both overweight/obese and the normal weight groups. Physical activity is known to improve insulin sensitivity^(38,39)^, including in obese individuals^(40)^. Furthermore, it lowers blood pressure and improves lipid profile^(41,42)^. It is also possible that physical activity is a marker of a generally healthy lifestyle.

Our data show that there was a higher alcohol consumption in the metabolically healthy group compared with the metabolically unhealthy group in both overweight/obese and in normal weight individuals. While alcohol raises serum triglycerides^(43)^, various authors have reported that alcohol consumption is associated with increased HDL levels^(43,44)^ and decreased prevalence of the metabolic syndrome^(45)^. In a 6-year longitudinal study, Huang *et al.*^(46)^ reported that alcohol consumers had a slower rate of age-related decline in HDL. Furthermore, alcohol consumption has been reported to be associated with lower LDL^(46)^, which would be expected to improve cardiovascular risk although this is not captured by the definition of cardiometabolic health. Justice *et al.*^(47)^ demonstrated that exposure of Wistar rats to alcohol for 3 months not only resulted in higher HDL and in lower total cholesterol and oxidised LDL, but that there was a significant reduction in the expression of hydroxymethylglutaryl-coenzyme A reductase (the rate-determining step in cholesterol synthesis) and in sterol regulatory element-binding protein-2 (a key transcription factor in cholesterol synthesis), as well as in up-regulation of paraoxonase-1 (which inhibits LDL oxidation). These observations suggest that alcohol may improve lipid profile via down-regulation of genes involved in cholesterol synthesis and up-regulation of genes that protect against LDL oxidation.

Smoking was more prevalent in metabolically unhealthy overweight/obese subjects than their metabolically healthy counterparts. While it is possible that smoking is acting as a marker for an unhealthy lifestyle, it is also possible that it is causally related to metabolic derangement. Smoking causes IR^(48)^ and also results in an adverse lipid profile^(49)^. However, there was no difference in smoking prevalence between metabolically healthy and unhealthy individuals in the normal weight category. Normal weight in these individuals may be due to smoking related loss of muscle mass^(50)^, rather than lack of adiposity, and which has been associated with onset of IR.

There was a lower proportion of MHO subjects with high fasting plasma glucose (>7⋅3 mmol/l), hypertriglyceridaemia (>1⋅7 mmol/l) or low HDL (<1⋅29 mmol/l in females and < 1⋅03 mmol/l in males) when compared with metabolically unhealthy normal weight subjects, in spite of a higher BMI. These findings are not unexpected since these parameters are used to categorise such individuals. However, they also had a lower VAI and higher ThC, both of which are known to be associated with decreased cardiovascular risk. On the other hand, they had similar waist, hip and neck circumferences, waist–hip ratio and IR (as measured by HOMA-IR). These findings suggest that the condition of MHO is not totally benign and that its risk probably lies somewhere between that of metabolically healthy normal weight and that of metabolically unhealthy normal weight categories. These observations have been consolidated in longitudinal studies that looked at incidence of type 2 diabetes and cardiovascular disease after a number of years of follow-up. For instance, Meigs *et al.*^(51)^ noted that after 11 years of follow-up, obese insulin-sensitive individuals had a 3-fold higher risk of developing type 2 diabetes when compared with insulin-sensitive normal weight individuals but were at lesser risk when compared with obese insulin-resistant subjects. Additionally, insulin-resistant normal weight individuals had a higher risk of developing type 2 diabetes when compared with insulin-sensitive obese individuals. These observations further support the possibility that the MHO phenotype is not totally benign but its risk is intermediate between the MHNW and MUHNW phenotypes. Furthermore, the meta-analysis by Kramer and colleagues found that healthy obese individuals and metabolically unhealthy subjects (irrespective of BMI) are at increased risk of all-cause and cardiovascular mortality when compared with healthy normal weight individuals and that the unhealthy normal weight phenotype carried the same risk for these events as that of the metabolically unhealthy obese cohort^(52)^. All things considered, this suggests that both the MUHNW and MHO phenotypes are not without risk and that the risk conferred by the MUHNW phenotype is similar to that of the highest risk group (the MUHO category), implying that attention should also be given to such individuals in terms of risk factor management.

To the authors’ knowledge, this is the first study in Malta that aimed to identify the prevalence of the different body composition phenotypes in such a well-characterised population. To date, little was known about the characteristics of the MHO and MUHNW phenotype in the Maltese Islands.

The present study has some limitations. Although it is acknowledged that there were more females than males in the studied population, other studies did not observe any heterogeneity between the genders in body composition between MHO and MUHO individuals^(53,54)^. Also, while standard definitions of metabolic health and IR were used to characterise the metabolically healthy from unhealthy phenotypes, data pertaining to proinflammatory cytokines, cardiorespiratory fitness or diet intake have not been studied. Furthermore, BMI was used as an index of obesity measurement, and thus, it could have misclassified individuals with short stature or muscular build. We chose the presence of one or less cardiometabolic abnormality to define metabolically health. Unfortunately, to date, no consensus exists in the scientific literature^(55)^, with as many as thirty definitions being used^(22)^. However, we used this cut-off as it is the most widely used, including landmark studies showing a relationship with all-cause and cardiovascular mortality as well as incident cardiovascular disease^(2,56,57)^. Furthermore, a large study found that metabolic health defined as ≤1 cardiometabolic parameters to be associated with increased cardiorespiratory fitness^(58)^.

The merits of the present study include a well characterised and an adequately sized representative sample of middle-aged adult subjects across the Maltese Islands. Survival bias is unlikely to be important in this age group. We have data on a number of important body composition parameters and lifestyle factors. We measured body composition parameters and blood markers directly, rather than rely on retrospectively collected data. Standard methods for data collection and for definition of metabolic health were used as already validated in previous studies and biochemical parameters were centrally analysed under appropriate quality controls. Another strength is that all data was collected by one individual, avoiding inter-observer variability.

## Conclusion

We found a high prevalence of the metabolically unhealthy phenotype in this middle-aged population which will likely result in significant future cardiovascular disease burden, with associated health economic implications. Metabolic health was associated with lower BMI, WC and neck circumference; more favourable biochemical parameters, physical exercise, alcohol consumption and less smoking. Various mechanisms may mediate these associations. Our data confirm the utility of a number of derived indices in predicting metabolic health including the WI, WHR, WHtR, WThR, BAI, VAI, CI, AVI, BRI and ABSI. However, thigh and arm circumferences were not useful in our cohort. More prospective studies which aim to identify the influence of genetic predisposition factors and early life/maternal characteristics are required in order to have a better understanding of the different body composition phenotypes in the Maltese Islands.
